# Maternal High-Fat Diet during Pregnancy and Lactation Influences Obestatin and Ghrelin Concentrations in Milk and Plasma of Wistar Rat Dams and Their Offspring

**DOI:** 10.1155/2016/5739763

**Published:** 2016-03-31

**Authors:** Monika Słupecka, Katarzyna Romanowicz, Jarosław Woliński

**Affiliations:** Department of Endocrinology, The Kielanowski Institute of Animal Physiology and Nutrition, Polish Academy of Sciences, 05-110 Jabłonna, Poland

## Abstract

The study aims to establish the effect of a maternal high-fat diet on obestatin concentration, total ghrelin, and ghrelin/obestatin ratio during pregnancy and lactation of Wistar rats and their offspring in the first 21 days of life. On the mating day, females were randomly allocated and fed either a high-fat diet (30% of fat; HF) or breeding diet (5% fat; BD) till the 21st day of lactation. Hormones were analyzed in the blood plasma and milk of rat dams as well as in the blood plasma of their offspring. HF resulted in a significant decrease in obestatin level on the 14th day of lactation and elevation on the 21st day. Plasma obestatin in HFD offspring was significantly higher than in BD ones. HF diet did not significantly affect dam plasma ghrelin until the 21st day of lactation. The ghrelin concentrations in milk after both diets were significantly lower than in blood plasma. Milk ghrelin in HF dams was significantly higher than in the BD ones. Plasma ghrelin from HF offspring was significantly higher than that from BD dams. Our results demonstrate that a maternal HF diet during pregnancy and lactation influences ghrelin and obestatin level in both dams and their offspring.

## 1. Introduction

Development and growth during the early life period are influenced by maternal health and diet composition [1]. Evidence from both epidemiological and animal model studies indicates that maternal diet influences metabolic status and plays a crucial role in the development of metabolic functions in offspring and their susceptibility to metabolic diseases in adulthood. Increased fat-consumption, an attribute of the Western diet, is considered a major triggering factor of metabolic impairments such as obesity, type II diabetes, insulin resistance, dyslipidemia, and hypertension. Studies in rats show that a high-fat diet during pregnancy and lactation has a marked impact on offspring body composition, risk of metabolic syndrome [2], and the development of obesity in both early life and adulthood [3, 4]. Moreover, it was shown that even mild maternal overnutrition caused by a high-fat diet led to increased adiposity [5], glucose intolerance [5], and altered brain appetite regulators in offspring [6, 7].

Ghrelin and obestatin are gastrointestinal peptides involved in the regulation of metabolic functions in rodents and humans. Although both peptides derive from a common preprohormone, a different posttranslation processing determines their functions which are reported to be opposite to several physiological functions. Moreover, several findings on obestatin are controversial as subsequent studies have provided contradictory results on its role in the regulation of food intake and body weight, energy expenditure control, and growth hormone secretion [7–9]. The nature of the obestatin receptor has also remained an open question. The initially proposed G-protein-coupled receptor GPR39 has been questioned due to a series of studies, which failed to demonstrate the ability of obestatin to bind to and activate this receptor [10–12]. Although obestatin's role in the control of metabolism is still unclear, the previous studies showed that the ghrelin/obestatin balance is essential for modulating energy homeostasis and adapting the organism to nutritional challenges. For example, deregulation in the ghrelin/obestatin ratio was observed in anorexia nervosa [13] and obesity, in both childhood [14] and adulthood [15]. Substantial amounts of ghrelin and obestatin were previously reported in the colostrum and human breast milk [16]. Obestatin and ghrelin as milk-contained bioactive peptides which can modulate energy homeostasis may play an important role in metabolism programming of newborns. However, the relationship between different maternal diets and obestatin and ghrelin balance during gestation and lactation of rats has not been examined yet. The present study aims both to explain how the concentration of stated hormones changes when diets varying in fat content will be given to rats during pregnancy and lactation and to learn whether they influence the plasma level of these two hormones in offspring until weaning time.

## 2. Materials and Methods

### 2.1. Animals

The experiments and treatments were conducted in compliance with European Union regulations concerning the protection of experimental animals. The Local Animal Ethics Committee located in Warsaw approved the study protocol. Sixteen adult female and sixteen male Wistar Han rats (11 weeks old) were obtained from the Center of Experimental Medicine at the Medical University of Bialystok, Poland. After two weeks of acclimatization, the females were examined with a vaginal impedance checker (Muromachi Kikai Co., Ltd.) for the precise determination of the stage of estrus for mating time. Based upon these measurements, female rats at the appropriate point in the estrous cycle were selected for breeding. The mating day was also a day when the animals were randomly allocated for either a high-fat diet (HF, 30% fat; 4.7 kcal/g; *n* = 6) or standard breeding diet (BD; 5% fat; 3.1 kcal/g; *n* = 6) (diets were purchased from Wytwórnia Pasz Morawski (Poland)). Main components of diets given to rats were presented in Table 1. Porcine lard was used as the source of fat in the HF diet. The following morning, mated females were examined for the presence of vaginal plug, and the day the plug was observed was considered as day 1 of gestation. On day 14 of gestation, the females were sampled for blood, weighed, and separated from the males. The day when females gave birth was considered day 1 of lactation. Only females who gave birth 21 ± 1 days after mating were chosen for the experiment (*n* = 6 for each diet). On the first day of lactation, the litters were standardized to ten pups. The supernumerary pups were sampled for blood and euthanized (day 1, HFO *n* = 7; BDO *n* = 5). On day 14 of lactation, two pups from each litter were sampled for blood and then sacrificed (*n* = 12 for each maternal diet). The rest of the pups stayed with their dams and continued to be used in the experiment till the 21st day of lactation when they were blood-sampled. Then all pups and rat dams (*n* = 6 for each diet) were sacrificed. Two pups from each litter were taken for further blood sampling (*n* = 12 for each maternal diet) while the rest were taken for another experiment. Starting from day 1 till day 21 of lactation all pups were blood-sampled via cardiac puncture under isoflurane or CO_2_ inhalation and then euthanized by overdosing of isoflurane (Baxter, USA) (day 1) or CO_2_ (days 14, 21 and rat dams).

### 2.2. Sampling Blood and Milk

Blood sampling was done on day 0 just before mating, on 14th-15th day of gestation, and on 14th and 21st days of lactation during the light phase between 11.00 and 11.30 a.m. (resting phase for rodents), but not during the dark period (active feeding phase), as the plasma ghrelin level has been shown to be altered by food intake [17].

Blood was sampled from the pups on the 1st (within 24 h after birth), 14th, and 21st days of life. Milk was sampled on 14th and 21st days of lactation before the blood sampling. In brief, 30 min before sampling, rat dams were separated from their pups and an intramuscular injection of 5 IU of oxytocin (Oxytocinum synt. 10 IU/mL, Biowet Puławy, Poland) was performed. Around 1–1.5 mL samples of milk were obtained from different randomly selected teats from each rat. Blood samples from the dams were collected from a tail vein. Just before the collection, the tails were heated in a water bath (40°C) to increase blood flow. Blood from the neonates and dams on the 21st day of lactation was collected via cardiac puncture.

The blood glucose levels were detected from a tail vein using Contour® Link (Bayer, USA).

Blood and milk samples were withdrawn on EDTA and aprotinin (0.6 TIU/mL of blood) and immediately centrifuged at 1.600 ×g for 15 minutes at 4°C. Blood plasma was harvested, distributed into Eppendorf tubes, deep frozen (−80°C), and stored until analysis. Before the milk samples were distributed into Eppendorf tubes and deep frozen (−80°C), the fat layer on the top was discarded.

Concentration of blood plasma triglycerides was determined spectrophotometrically (MAXMAT PL multidisciplinary diagnostic platform, Erba Diagnostics France SARL, France) using ELITech ready-to-use reagents (ELITech Group, France) according to method described previously [18].

### 2.3. Obestatin and Ghrelin Radioimmunoassay

Rat milk and plasma samples were assayed for obestatin and total ghrelin (acyl and nonacyl) concentration using commercially available radioimmunoassay (RIA) kits: Ghrelin (Rat, Mouse) RIA Kit and Obestatin (Rat, Mouse) Ultra-Sensitive RIA Kit (Phoenix Pharmaceuticals, Inc., USA) according to the manufacturer's instructions. Spike and recovery of obestatin and total ghrelin were measured in three different plasma or milk samples. The assays were performed in one run. The mean percent recovery for obestatin and ghrelin in plasma was 90% and 95%, respectively. The interassay coefficient of variation (CV) for obestatin and ghrelin in plasma was 6.6% and 6.0%, respectively. Before the assays, the milk samples were sonified for 15 min. Mean percent recovery was 90% for both obestatin and ghrelin in milk. The interassay CV for obestatin and ghrelin in milk was 7.6% and 5.6%, respectively.

### 2.4. Statistical Analysis

The data are expressed as means ± SEM. One-way analysis of variance (ANOVA) followed by post hoc Tukey-Kramer or Kruskal-Wallis test followed by post hoc Dunn's Multiple Comparison Test or regular two-way ANOVA was used to determine statistically significant differences between the groups' tested time points or/and different samples (Prism 5 for Mac OS X, Version 5.0d, Graph Pad Software, San Diego, CA, USA). The Pearson test was used for correlation analysis. In all statistical analyses, *p* < 0.05 was taken as the level of significance.

## 3. Results

### 3.1. Body Weights, Blood Glucose, and Plasma Triglycerides

Body weights of rat females from mating until 21st day of lactation, the blood glucose level, and plasma triglycerides during tested time points were presented in Table 2(a). No differences in body weight between BD dams and HF dams were observed. The level of plasma triglycerides was unchanged in pregnancy but during lactation (14th and 21st day) increased significantly in HF dams. Feeding dam rats with high-fat diet during pregnancy and lactation resulted in significant increase in body weight and plasma triglycerides of their offspring (HFO) starting from the 14th day of life as compared to control pups (BDO) (Table 2(b)). Maternal diet had no effect on the litter size (data not shown) and the level of blood glucose in rat neonates (Table 2(b)).

### 3.2. Obestatin in Blood Plasma of Rat Dams and Pups and in Maternal Milk

**Figure 1:**
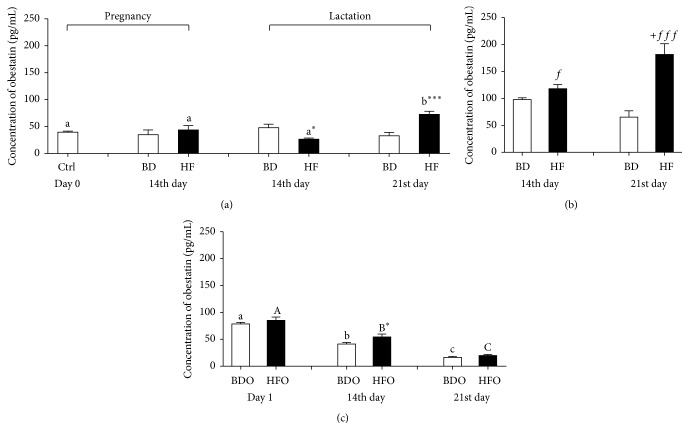
Obestatin concentration (pg/mL) in blood plasma (a) and milk (b) of female rats fed with standard breeding diet (BD) or high-fat diet (HF) in pregnancy and lactation during different reproductive stages (before mating, day 0, 14th day of pregnancy, and 14th and 21st day of lactation) and blood plasma of pups from BD dams (BDO) or HF dams (HFO) in the first 21 days of life (c). Results are presented as means ± SEM. ^a,b,c^Statistical differences in obestatin plasma concentration between days within control treatment (BD or BDO). ^A,B^Statistical differences in obestatin plasma concentration between days within HF treatment and HFO treatment. ^+^Statistical differences between BD and HF group and between BDO and HFO group (*p* < 0.05). ^+^Statistical differences in obestatin milk concentration between days within treatment (*p* < 0.05). *f* indicates statistical differences in milk obestatin concentration between BD and HF group (^*f*^ *p* < 0.05, ^*fff*^ *p* < 0.001).

In comparison with that in nonpregnant female rats (day 0), the maternal obestatin concentration in plasma of BD rats was unchanged in pregnancy (14th day of gestation) and in tested time points during lactation (14th and 21st day). In contrast, the HF diet during pregnancy and lactation resulted in a significant decrease in the obestatin level on 14th day of lactation and significant elevation on 21st day in comparison with both nonpregnant females and lactating BD females (Figure 1(a)). The obestatin concentration in milk in 14th and 21st day of lactation was significantly higher than in the blood plasma of rat dams. In control rat dams (BD group), concentration of obestatin in milk did not differ significantly in tested time points during lactation, whereas the HF diet resulted in a significant elevation in milk obestatin as compared to the BD diet, where peptide concentration rising significantly from day 14 till 21st day of lactation (Figure 1(a)). A relationship between the obestatin concentration in milk and maternal blood in 14th day of lactation was observed in both diets (Table 3(a)). In the BD group, we observed a negative correlation (*r* = −0.838, *p* = 0.038), whereas in the HF rat dams we found a strong positive correlation (*r* = 1.000, *p* < 0.0001). Significant negative correlations were observed between maternal plasma obestatin and ghrelin for BD rats in 14th and 21st day of lactation and for HF rats in 14th day of pregnancy and lactation (Table 3(a)). Interestingly, positive relationships between these hormones in milk were observed in the 14th day of lactation for BD dams (*r* = 0.859, *p* = 0.014) and in the 21st of lactation for HF dams (*r* = 0.720, *p* = 0.014) (Table 4(a)).

Also a relationship between the body weight of dams and the obestatin concentration in blood plasma in 21st day of lactation was observed on both diets. The positive correlation was observed between the body weight of BD dams and plasma blood obestatin concentration in the 21st day of lactation (*r* = 0.976, *p* = 0.002), whereas in the HF dams we found a negative correlation (*r* = −0.881, *p* = 0.010) (Table 3(a)). No correlation was found between the glucose concentration in maternal blood and the obestatin level in either blood or maternal milk (Table 3(a)).

The highest obestatin concentration in the blood plasma of offspring from control rat dams (group BDO) was observed on day 1 of lactation and then the significant decrease was observed in the following days of lactation (Figure 1(c)). In pups from HF rat dams (HFO), the obestatin concentration was higher in tested time points (significant difference in the 14th day of life, *p* = 0.032) but the same dependence was observed in the form of a significant decrease in hormone level in the successive days of life. No correlations were found between plasma obestatin and ghrelin in offspring on either treatment (Table 4(b)).

### 3.3. Ghrelin in Blood Plasma of Rat Dams and Pups and in Maternal Milk

**Figure 2:**
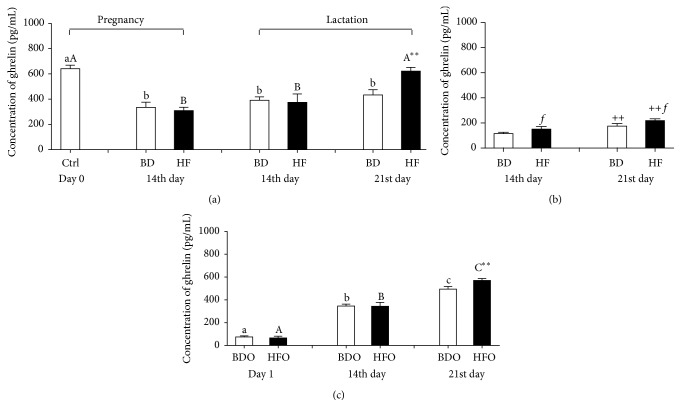
Ghrelin concentration (pg/mL) in blood plasma (a) and milk (b) of female rats fed with standard breeding diet (BD) or high-fat diet (HF) in pregnancy and lactation during different reproductive stages (before mating, day 0, 14th day of pregnancy, and 14th and 21st day of lactation) and blood plasma of pups from BD dams (BDO) or HF dams (HFO) in the first 21 days of life (c). Results are presented as means ± SEM. ^a,b,c^Statistical differences in ghrelin plasma concentration between days within control treatment (BD or BDO). ^A,B,C^Statistical differences in ghrelin plasma concentration between days within HF treatment and HFO group. ^*∗*^Statistical differences between BD and HF group and between BDO and HFO group ^*∗∗*^ *p* < 0.01. ^+^Statistical differences in ghrelin milk concentration between days within treatment (^++^ *p* < 0.01). *f* indicates statistical differences in ghrelin milk concentration between BD and HF group (^*f*^ *p* < 0.05).

In the BD rat dams, the ghrelin concentration in blood plasma decreased in pregnancy and lactation period in comparison to nonpregnant females (day 0) (Figure 2(a)). On the 21st day of lactation, a significant increase in ghrelin concentration in HF dams was found (*p* = 0.005) in comparison to BD ones. The observed level of ghrelin in this time point did not differ (*p* = 0.705) from the basal level for ghrelin in rat dams before mating (Figure 2(a)). The ghrelin concentrations in maternal milk from both studied groups of rat dams were significantly lower than in blood plasma and grew significantly in the subsequent days of lactation. The milk ghrelin concentration in HF dams was significantly higher than that in the BD animals (Figure 2(b)). Negative correlations were found between blood plasma and milk ghrelin in the control rat dams on the 21st day of lactation (*r* = −0.737, *p* = 0.029) and between body weight and the plasma ghrelin concentration on the 14th day of pregnancy in the HF rat dams (*r* = −0.903, *p* = 0.048). By contrast, a positive correlation was found between blood plasma ghrelin and glucose on the 21st day of lactation (*r* = 0.852, *p* = 0.033) (Table 3(b)).

The ghrelin blood plasma concentration in neonates increased significantly in the subsequent days of life. On the 21st day of lactation, the ghrelin concentration in the blood plasma of neonates from the HF dams was significantly higher than in neonates from the control (BD) rat dams (Figure 2(c)).

### 3.4. Ghrelin/Obestatin Ratio in Blood Plasma of Rat Dams and Pups and in Maternal Milk

**Figure 3:**
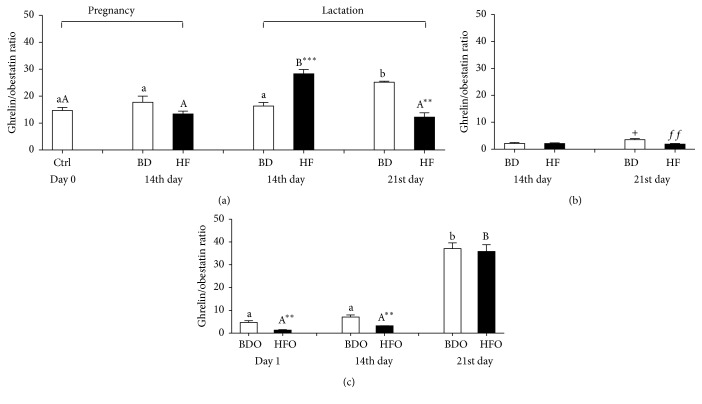
Ghrelin/obestatin ratio in blood plasma (a) and milk (b) of female rats fed with standard breeding diet (BD) or high-fat diet (HF) in pregnancy and lactation during different reproductive stages (before mating, day 0, 14th day of pregnancy, and 14th and 21st day of lactation) and blood plasma of pups from BD dams (BDO) or HF dams (HFO) in the first 21 days of life (c). Results are presented as means ± SEM. ^a,b^Statistical differences in ghrelin/obestatin plasma concentration between days within control treatment (BD or BDO). ^A,B^Statistical differences in ghrelin/obestatin plasma concentration between days within HF treatment and HFO group. ^*∗*^Statistical differences between BD and HF group (^*∗∗*^ *p* < 0.01, ^*∗∗∗*^ *p* < 0.001). ^+^Statistical differences in ghrelin/obestatin milk concentration between days within treatment (^+^ *p* < 0.05). *f* indicates statistical differences in ghrelin/obestatin milk concentration between BD and HF group (^*ff*^ *p* < 0.01).

The ghrelin/obestatin ratio for the control maternal plasma was stable during the tested time points except for a peak on the 21st day of lactation when we observed both a significant elevation in this parameter and a difference with HF dams. In the HF dams, the ratio was unchanged except on the 14th day of lactation when the ratio went up which resulted in a significant difference with BD dams (Figure 3(a)).

On the 21st day of lactation, the ghrelin/obestatin ratio significantly decreased in milk from experimental dams (HF) in comparison to control (BD) rat dams (Figure 3(b)). In plasma neonates from the control dams (BDO), the ratio was stable during the first fourteen days of life and then increased significantly on the 21st day of life (*p* < 0.0001). The same pattern was observed in neonates from the experimental dams (HFO), but the values for the ghrelin/obestatin ratio in the first fourteen days of life were significantly lower (*p* = 0.006; *p* = 0.010, resp.) than in those in the control neonates (Figure 3(c)).

## 4. Discussion

Our study reports on the concentrations of ghrelin and obestatin in the blood of rat dams, fed diets differing in fat content during pregnancy and lactation, together with their concentrations in the milk during lactation and blood of offspring. We reported that a high-fat diet during pregnancy and lactation did not change the ghrelin plasma pattern on the 14th day of pregnancy and lactation. However, on the 21st day of lactation the ghrelin concentration in HF dams was elevated in comparison to that in the BD animals. The effect of HF diet consumption on ghrelin concentration is not conclusive as both no changes [19, 20] and the significant decrease [21, 22] of this hormone have been observed in rodents. In our study, the ghrelin concentration in HF dam rats could be additionally associated with specific metabolic changes that occur during pregnancy and lactation. Previous studies revealed that the energy demands for lactation in rodents can exceed 60% resulting in a threefold increase in food intake [23]. However, reduced food intake and greater weight loss were shown in obese rats in the first days of lactation [23, 24]. Keesey and Hirvonen [25] proposed the existence of a “body weight set point” in rodents and humans so that body weight decrease or increase is corrected by altering food intake and energy expenditure to maintain the target body weight. Although this weight-control mechanism was not investigated in the present study, it could explain the lack of effects on dam body weight depending on the diet which was reported in our study as well as in other studies on rodents fed with HF diet during gestation and lactation [26–28]. This phenomenon may be also explained by results showing that fat mobilization slightly contributes to total milk energy output and that larger milk fat production in obese rats is achieved by the mobilization of triglycerides from adipose tissue and larger plasma lipid extraction rather than increased food intake. In our study we did not measure the daily food intake; however we observed the increase in milk fat content on the 14th and 21st day of lactation (data not shown) together with significantly higher concentration of plasma triglycerides of HF rats starting from the 14th day of gestation till the 21st day of lactation. Increased lipolysis during lactation in HF rats may be associated with an observed decrease in the concentration of plasma obestatin on the 14th day of lactation and then its significant increase at the end of lactation (21st day) although recent studies by Pruszyńska-Oszmałek et al. [29] have suggested that obestatin may inhibit lipogenesis. It should also be mentioned that, in our study, rats were fed a HF diet for a relatively short period of six weeks. Moreover, in our model we compared a high-fat diet (HF) together with a standard breeding diet (BD), which was formulated to fulfill higher energetic demands of rat dams during gestation and lactation, so it is more energetic than standard rodent chow.

We have also found that the ghrelin/obestatin ratio increased in the plasma of HF dams on the 14th day of lactation and then significantly decreased on the 21st day. Previous studies on humans showed that the ghrelin/obestatin ratio was lower in obese subjects and negatively correlated with BMI and indices of abdominal fat distribution [15]. This finding seems to support the concept that, during lactation, a HF diet leads to a negative energy balance (e.g., increased lipolysis) and then when lactation ends (on the 21st day) the HF diet starts to develop overweight or/and obesity.

Although we did not observe any changes in the body weight of dams depending on the diet, we reported changes in the hormones concentration to occur in the milk and plasma of neonates. Both tested hormones were significantly elevated in the milk of HF dams and resulted in a tendency to increase their plasma concentrations in neonates. On the 14th day of lactation, this increase was significant for obestatin concentration in the plasma of offspring from HF dams (HFO), and on the 21st day of lactation we observed a significant elevation in the ghrelin level in HFO. These results suggest that in rats the crucial metabolic changes occur from the 14th day of life. A higher concentration of ghrelin, an orexigenic hormone [30], in blood at weaning suggests that offspring from HF dams may be programmed by ghrelin to take in more food from an early age. Moreover, mothers' milk may be an important factor in this programming. The intestine of a rat remains open to the uptake of maternal milk-derived macromolecules until the age of 21 days, when intestinal permeability decreases to the adult level [31]. However, in our studies the relationships between maternal hormones in milk and their concentration in the blood plasma of their offspring were observed only for ghrelin in HFO on the 14th day of lactation. Our study shows that the concentrations of both studied hormones increased due to the maternal high-fat diet, which raises the question of its possible effect on neonate development. It was previously shown that gut peptides may be involved in the pathways affecting both appetite and other complex signaling pathways within the brain which may potentially affect behavioral and homeostatic processes beyond appetite regulation. Further studies are needed.

We also reported that the maternal high-fat diet resulted in changes in the ghrelin/obestatin ratio in both rat dams and their offspring. Vicennati et al. [15] observed a lower ghrelin/obestatin ratio in the blood plasma of obese women and suggested that disparate changes in circulating ghrelin and obestatin level represent adaptive modifications to obesity development rather than primary defects and that their alteration in circulating blood levels reflects an imbalance of regulatory factors or mechanisms responsible, in turn, for their metabolic processes and action. This hypothesis is in agreement with our findings as we observed a significant decrease in the abovementioned ratio in HF dams when lactation is ending and in their pups in the first 14 days of life.

Our results confirm studies in humans showing that the obestatin concentration in breast milk is over twofold higher than in maternal plasma [16]. These observations suggest that the mammary gland is a source of obestatin. Interestingly, the pattern of obestatin concentration in rat milk during lactation is opposite to its concentration in the blood plasma of neonates. Our results show that the highest plasma concentration of obestatin is observed in newborns compared to that during postnatal development decreases suggesting that milk-born obestatin could be an important source of this hormone in the very early stage of postnatal development. Although ghrelin and obestatin originate from the same precursor peptide, their developmental blood patterns in rat pups differ.

A plasma concentration of ghrelin in neonates increasing with age as well as peptide concentration in the blood plasma of dam rats during pregnancy and lactation had been previously reported [32, 33]. Similar to studies by Taylor et al. [32] we observed no changes in BD rat dams in ghrelin concentration between pregnancy and lactation. However, similar to results shown by Shibata et al. [33] we observed a significant decrease in plasma ghrelin concentration in pregnant (14th day of gestation) rats in comparison to rats in the proestrus stage of the estrous cycle, whereas Taylor et al. [32] did not report any significant changes.

In addition, studies on pregnant women have shown a significant decrease in ghrelin concentration during the third trimester as compared with nonpregnant women [19]. This suggests that a reduced plasma ghrelin level may have important implications for the course of pregnancy. Shibata et al. [33] reported that ghrelin release by the hypothalamus affected ghrelin plasma concentration during pregnancy instead of ghrelin production in the stomach.

## 5. Conclusions

In conclusion, this study shows that a maternal high-fat diet during pregnancy and lactation influences ghrelin and obestatin plasma levels in both dams and their offspring. Moreover, mother milk is an important factor for the transmission of information between mother and infant and the maternal dietary fat content influences its ghrelin and obestatin concentration.

## Figures and Tables

**Table 1:** Main components of diets given to rats.

Amount per kg	Standard breeding diet (BD, 5% fat)	High-fat diet (HF, 30% fat)
Dry matter	885 g	918 g
Crude protein	222 g	190 g
Crude lipids	50 g	297 g
Carbohydrates	572 g	401 g
Crude fiber	46 g	32 g
Crude ash	41 g	30 g
Metabolic energy	12,8 MJ/3057 kcal	19 MJ/4658 kcal

**(a):** 

	Mating		Pregnancy		Lactation			
	First d		14th d		14th d		21st d	
	BD	HF	BD	HF	BD	HF	BD	HF
Body weight (g)	246 ± 12	224 ± 6^A^	280 ± 14	259 ± 8^B^	285 ± 17	253 ± 6^B^	283 ± 19	253 ± 6^B^
Blood glucose (mmol/L)	7.38 ± 0.1^a^	8.27 ± 0.12^A^	6.88 ± 0.38^a^	7.16 ± 0.11^A^	11.44 ± 0.61^b^	8.61 ± 0.61^B*∗*^	11.33 ± 1.33^b^	9.88 ± 0.72^AB^
Plasma triglycerides (mmol/L)	1.1 ± 0.1^a^	0.8 ± 0.1^A^	2.7 ± 0.3^b^	3.2 ± 0.8^B^	1.0 ± 0.1^a^	2.9 ± 0.5^B*∗∗∗*^	1.3 ± 0.1^ab^	2.8 ± 0.5^B*∗∗*^

BD: females fed with standard breeding diet during pregnancy and lactation; HF: females fed with high-fat diet during pregnancy and lactation. Data are expressed as means ± SEM. ^a,b^Statistical differences between days within BD treatment. ^A,B^Statistical differences between days within HF treatment. ^*∗*^Statistical differences between BD and HF group (^*∗*^ *p* < 0.05, ^*∗∗*^ *p* < 0.01, ^*∗∗∗*^ *p* < 0.001).

**(b):** 

	First d		14th d		21st d	
	BDO	HFO	BDO	HFO	BDO	HFO
Body weight (g)	6.2 ± 0.2^a^	5.9 ± 0.25^A^	22 ± 0.3^b^	27 ± 0.3^B*∗∗∗∗*^	37 ± 1.3^c^	45 ± 1.1^C*∗∗∗*^
Blood glucose (mmol/L)	4.3 ± 0.6^a^	4.1 ± 0.4^A^	7.8 ± 0.4^ab^	7.4 ± 0.6^AB^	9.4 ± 1.2^b^	9.9 ± 2.0^B^
Plasma triglycerides (mmol/L)	1.7 ± 0.2^a^	1.0 ± 0.3^A*∗*^	0.6 ± 0.1^b^	2.1 ± 0.2^AB*∗∗∗∗*^	2.3 ± 0.4^a^	4.8 ± 0.8^B*∗*^

BDO: rat neonates from females fed with standard breeding diet during pregnancy and lactation; HFO: rat neonates from females fed with high-fat diet during pregnancy and lactation. Data are expressed as means ± SEM. ^a,b,c^Statistical differences between days within BDO treatment. ^A,B,C^Statistical differences between days within HFO treatment. ^*∗*^Statistical differences between BDO and HFO group (^*∗*^ *p* < 0.05, ^*∗∗*^ *p* < 0.001, ^*∗∗∗*^ *p* < 0.0001).

**(a):** 

	Pregnancy		Lactation			
	14th d		14th d		21st d	
	BD	HF	BD	HF	BD	HF
Plasma obestatin versus body weight	*r* = 0.319	*r* = 0.531	*r* = 0.158	*r* = 0.625	*r* = 0.976	*r* = −0.881
	*p* = 0.299	*p* = 0.139	*p* = 0.367	*p* = 0.187	*p* = 0.002^*∗∗*^	*p* = 0.010^*∗*^

Plasma obestatin versus milk obestatin	*r* = 0.547	*r* = 0.267	*r* = −0.838	*r* = 1.000	*r* = −0.310	*r* = −0.527
	*p* = 0.234	*p* = 0.312	*p* = 0.038^*∗*^	*p* < 0.0001^*∗∗∗∗*^	*p* = 0.306	*p* = 0.141

Milk obestatin versus newborns body weight			*r* = −0.653	*r* = −0.858	*r* = −0.185	*r* = 0.271
			*p* = 0.056	*p* = 0.007^*∗∗*^	*p* = 0.317	*p* = 0.210

No correlations were observed for following parameters: plasma obestatin versus blood glucose; plasma obestatin versus newborn plasma obestatin; milk obestatin versus body weight; milk obestatin versus blood glucose.

**(b):** 

	Pregnancy		Lactation			
	14th d		14th d		21st d	
	BD	HF	BD	HF	BD	HF
Plasma ghrelin versus body weight	*r* = 0.351	*r* = −0.903	*r* = −0.203	*r* = −0.738	*r* = −0.517	*r* = 0.451
	*p* = 0.325	*p* = 0.048^*∗*^	*p* = 0.371	*p* = 0.236	*p* = 0.117	*p* = 0.223

Plasma ghrelin versus milk ghrelin	*r* = 0.319	*r* = 0.228	*r* = 0.540	*r* = 0.978	*r* = −0.737	*r* = −0.021
	*p* = 0.311	*p* = 0.374	*p* = 0.230	*p* = 0.067	*p* = 0.029^*∗*^	*p* = 0.484

Plasma ghrelin versus blood glucose	*r* = 0.567	*r* = 0.104	*r* = 0.113	*r* = 0.478	*r* = −0.342	*r* = 0.852
	*p* = 0.219	*p* = 0.512	*p* = 0.412	*p* = 0.196	*p* = 0.254	*p* = 0.033^*∗*^

Milk ghrelin versus newborns plasma ghrelin			*r* = 0.463	*r* = 0.980	*r* = 0.045	*r* = 0.332
			*p* = 0.148	*p* = 0.010^*∗*^	*p* = 0.462	*p* = 0.174

No correlations were observed for following parameters: plasma ghrelin versus newborn plasma ghrelin, milk ghrelin versus body weight, milk ghrelin versus blood glucose, and milk ghrelin versus newborn plasma ghrelin. BD: females fed with standard breeding diet during pregnancy and lactation; HF: female rats fed with high-fat diet during pregnancy and lactation; ^*∗*^ *p* < 0.05, ^*∗∗*^ *p* < 0.05, ^*∗∗∗∗*^ *p* < 0.0001.

**(a):** 

	Pregnancy		Lactation			
	14th d		14th d		21st d	
	BD	HF	BD	HF	BD	HF
Dam plasma obestatin versus ghrelin	*r* = − 0.066	*r* = − 0.995	*r* = − 0.907	*r* = − 0.840	*r* = − 1.000	*r* = − 0.634
	*p* = 0.404	*p* = 0.002^*∗∗*^	*p* = 0.047^*∗*^	*p* = 0.037^*∗*^	*p* = 0.001^*∗∗*^	*p* = 0.088

Milk obestatin versus ghrelin			*r* = 0.859	*r* = − 0.087	*r* = − 0.035	*r* = 0.720
			*p* = 0.014^*∗*^	*p* = 0.445	*p* = 0.473	*p* = 0.014^*∗*^

BD: females fed with standard breeding diet during pregnancy and lactation; HF: female rats fed with high-fat diet during pregnancy and lactation; ^*∗*^ *p* < 0.05, ^*∗∗*^ *p* < 0.01.

**(b):** 

	1st d		14th d		21st d	
	BDO	HFO	BDO	HFO	BDO	HFO
Newborns plasma obestatin versus ghrelin	*r* = − 0.461	*r* = 0.614	*r* = − 0.720	*r* = − 0.510	*r* = 0.421	*r* = 0.252
	*p* = 0.217	*p* = 0.097	*p* = 0.053	*p* = 0.190	*p* = 0.087	*p* = 0.173

BDO: pups from females fed with standard breeding diet during pregnancy and lactation; HFO: pups from females fed with high-fat diet during pregnancy and lactation.
